# Efficacy of Single-Dose Radiotherapy in Preventing Posttraumatic Tendon Adhesion

**DOI:** 10.7759/cureus.8410

**Published:** 2020-06-02

**Authors:** Cenk Ermutlu, Tufan Kaleli, Ulviye Yalcinkaya, Sibel Cetintas, Teoman Atici

**Affiliations:** 1 Orthopaedics, Bursa Uludag University School of Medicine, Bursa, TUR; 2 Orthopaedics and Traumatology, Bursa Uludag University School of Medicine, Bursa, TUR; 3 Pathology, Bursa Uludag University School of Medicine, Bursa, TUR; 4 Radiation Oncology, Bursa Uludag University School of Medicine, Bursa, TUR

**Keywords:** animal study, experimental study, rabbit, radiotherapy, posttraumatic tendon adhesion, tendon adhesion

## Abstract

Background and Aim

Posttraumatic peritendinous adhesion is the greatest obstacle to achieve normal tendon function following lacerations of extrinsic flexor tendons of the hand. In this study, we aimed to evaluate whether single-dose radiotherapy (RT) has the potential to modulate intrasynovial tendon adhesions.

Materials and Methods

A total of 80 tendons from the third to fourth flexor profundus of both hind paws of 20 adult New Zealand rabbits were used in this study. Rabbits in the RT group received 3 Gy of X-irradiation in a single fraction. Histopathological evaluation of longitudinal sections of tendons was made using the Tang grading system for peritendinous adhesions. Intratendinous quality of the healing tissue in the laceration zone was assessed using a modified Movin scale.

Results

Adhesion and inflammatory response were greater in the RT group (p˂0.001). Tendon healing in the radiation group was found to be more uniform and organized compared with the control group. However, this difference was not statistically significant. The nuclei of the tenocytes in the radiation group showed a closer resemblance to normal tendon tissue when compared with the control group (p=0.007).

Conclusions

Despite RT’s certain advantages such as extracorporeal use, anti-inflammatory effect, and homogenous tissue penetration, 3-Gy X-irradiation resulted in increased peritendinous posttraumatic adhesion, possibly due to dose imbalance. Increased roundness in the tenocyte nuclei was present in the RT group. Studies with different dosing regimens and a higher number of subjects are necessary to establish an ideal dose suppressing the synovial response without compromising tendon healing.

## Introduction

Tendons are connective tissue elements that provide joint movement by transmitting muscle contraction to the bones. Because of the difficulties encountered in the treatment and the lack of satisfactory clinical results, primary repair of intrasynovial flexor tendon injuries was not recommended for a long duration of time and this anatomical region was named as “no man’s land” [1].

Adhesion between the area of injury and the surrounding tissues following tendon injuries is the greatest undesirable interference to the normal functioning of the tendon. Tendon healing is a multifactorial process with intercellular mediators and dense cell chemotaxis, of which different elements are targeted in order to prevent adhesions at different stages of tendon repair [2]. Low friction suturing techniques, early postoperative rehabilitation, use of physical barriers, antiadhesive pharmacological chemicals, agents effective on tendon surface lubrication enhancement, cell cycle, and apoptosis are among the suggested tools for the prevention of postoperative adhesion following the surgical treatment of flexor tendon injuries [3].

Radiotherapy (RT) has a wide area of application besides the management of malignant diseases, and it has been implemented in the management of a wide range of medical conditions including heterotrophic ossification after orthopedic surgery, Graves’ ophthalmopathy, keloid formation, and stenosed coronary stents [4]. Although the method of action for RT is the prevention of undesirable excess cell and tissue formation in response to different mediators, its efficiency on synovial fibroblasts and the management of tendon adhesion has not been evaluated so far.

In this experimental animal study, we aimed to evaluate the effectiveness of this treatment modality on the prevention of the development of posttraumatic tendon adhesion during the healing process following laceration of intrasynovial flexor tendon injuries.

## Materials and methods

Study subjects

The experiments were performed on 20 adult 2,400-3,800 gram New Zealand rabbits. The study was approved by the Uludag University Animal Experiments Local Ethics Committee and conducted according to the criteria set by the Declaration of Helsinki. Sample size determination was performed using nonparametric analysis on Tang scoring prior to the study with a set study power value of 0.85. Considering that four tendon samples will be obtained from each animal, it was calculated that the study groups should consist of a minimum of five rabbits. Owing to the possible loss of subjects after surgery and the tendon destruction during the longitudinal dissection process for the preparation of the histopathological samples, the study groups were established as 10 animals per group.

Surgical technique

**Figure 1:**
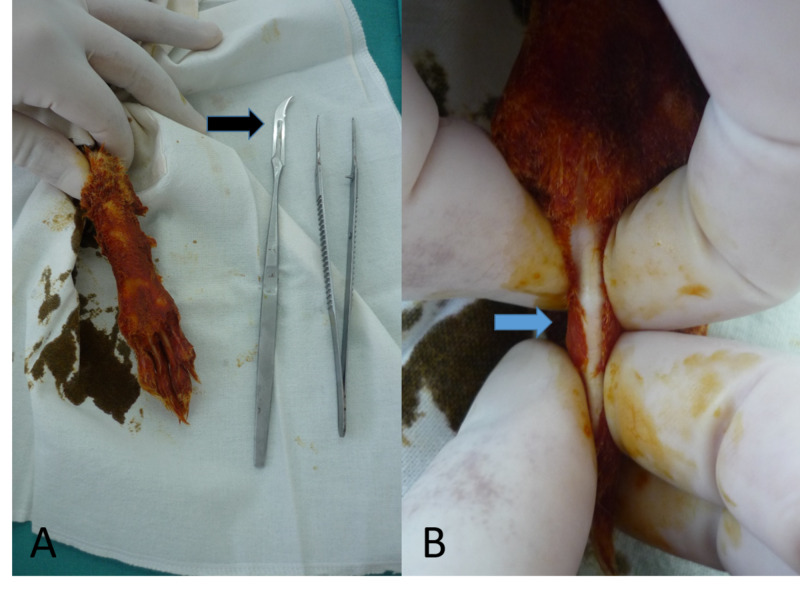
The preparation phase for the surgical procedure. (A) Preparation of the plantar side of the rabbit’s paw. (B) By pulling the skin of the third phalanx to the sides, the flexor tendon and the pulley become visible under the thin, intact skin.

**Figure 2:**
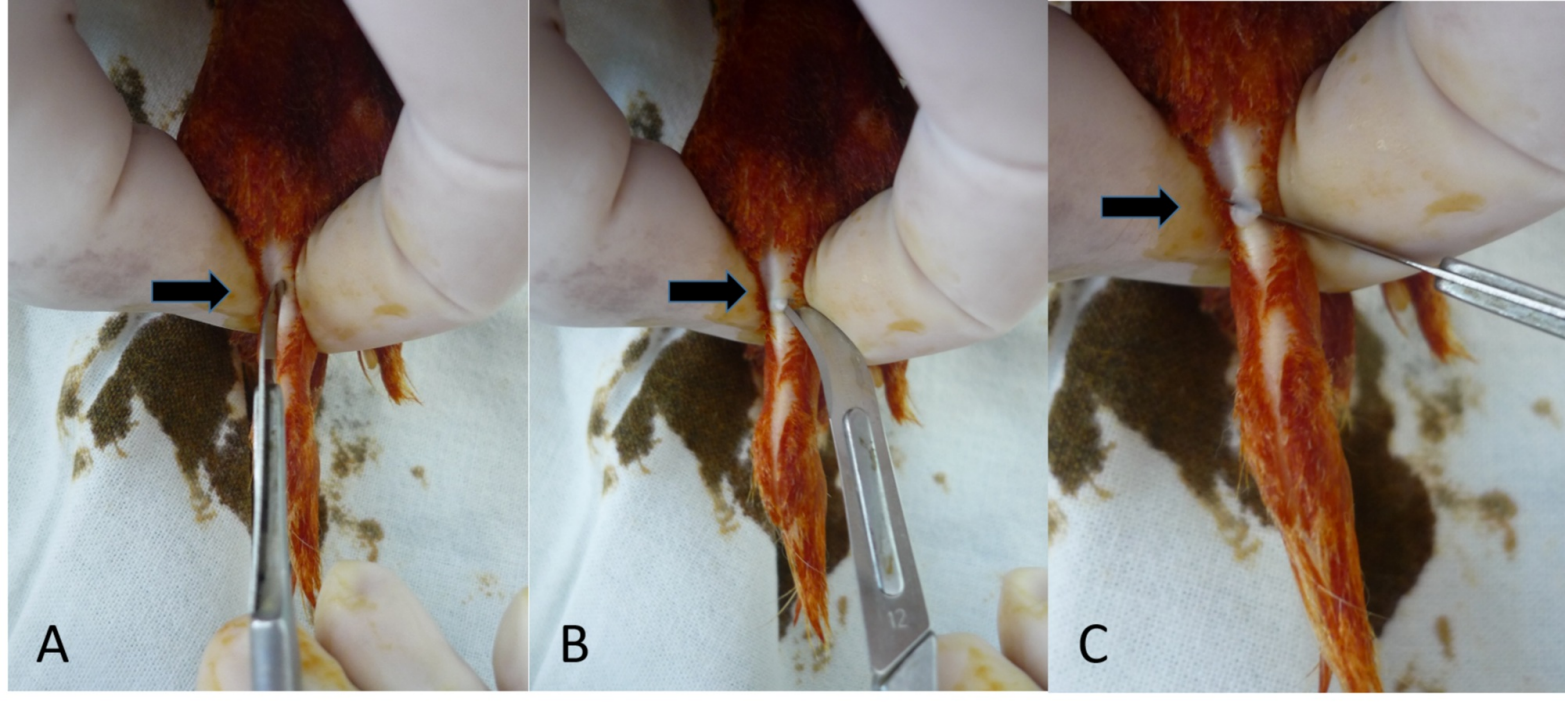
Surgical procedure for the experiment. (A) Tendon is incised longitudinally in the midline with the tip of a no. 12 blade. (B,C) The curved blade is rotated 90 degrees, and a partial tenotomy is made.

**Figure 3:**
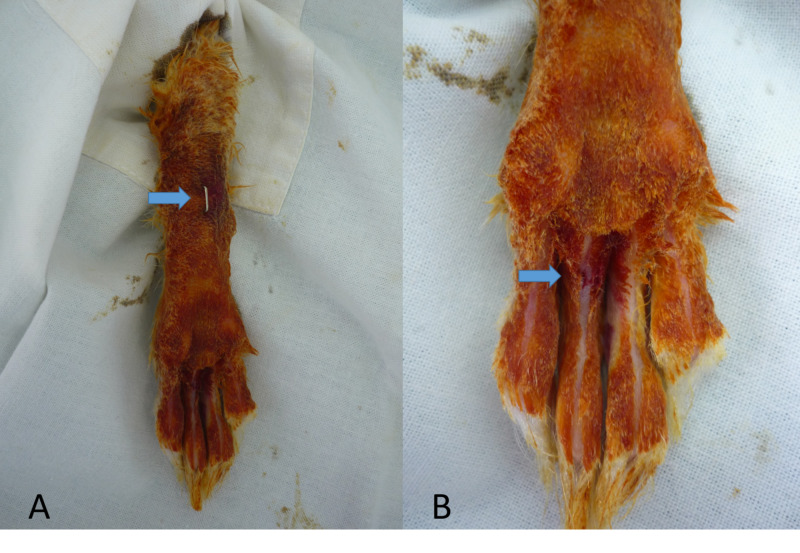
The postprocedural phase of the experiment. (A) Common flexor tendons are cut through a plantar incision to facilitate immobility and adhesion. (B) The phalanx after the procedure.

Anesthesia of all subjects was provided by intramuscular (IM) injection of 30 mg/kg of ketamine hydrochloride and 4 mg/kg of xylazine hydrochloride. Each rabbit received IM antibiotic (cefazolin sodium 15 mg/kg) for surgical prophylaxis. Following the surgical area preparation using a 10% povidone-iodine solution (Batidex, Cimedis, Ankara, Turkey), the paws were shaved for the surgery in the prone position. The surgical area was covered with sterile drapes, and the surgical procedure was performed at the level of the third and fourth flexor profundus tendons of the hind paws. By pulling the thin skin to the sides, the tendons and the pulley became visible on the plantar side of the phalanx despite an intact skin. Tip of a no. 12 blade was placed on the midline of the flexor profundus tendon in line with the phalanx’s axis and the tendon tissue was pierced till reaching the phalanx. The curved blade was rotated 90 degrees, and a partial tenotomy was performed. Releasing the retracted skin fully covered the small hole caused by the blade tip, and no suture was necessary. Common flexor tendons were cut through a plantar incision to facilitate immobility and adhesion formation. The plantar incision was closed with a single staple (Figures 1-3).

On the first postoperative day, 2 mg/mL of acetaminophen was added to drinking water for postoperative analgesia. None of the subjects received antibiotics following the surgery.

Radiotherapy

A preliminary study was performed on five animals in order to determine the radiation dose in the study. After the animals were anesthetized, each subject was subjected to five different doses (3 Gy, 6 Gy, 10 Gy, 15 Gy, 20 Gy) of ionizing radiation. Histopathological examination of the subjects that received radiation at 6 Gy or greater doses resulted in local necrosis and bleeding zones between the bone trabeculae structure. The radiation dose was decided to be set at 3 Gy considering the experimental suitability and efficiency.

Twenty subjects underwent partial tenotomy with the same anesthetic and surgical technique applied to the preliminary study group. The subjects were divided into two groups as control and RT groups with equal gender distributions (5 males and 5 females in each group). Ten animals in the RT group were admitted into the RT room after being anesthetized with the same anesthesia method applied for the surgery. The rabbits were placed feet-first prone on the study table, with the palm of the paws facing upward. The field size was adjusted to 10 x 10 cm, and the radiation dose was calculated using pencil beam algorithm. Calculation was specified for the mid-point of the limb, and a 0.5-cm gel bolus was placed on the target zone. No field verification was performed for the individual test subjects. However, routine calibration of the linear accelerator was performed on a weekly and daily basis. The extremities of the subjects were irradiated at a dose of 3 Gy using a linear accelerator (Mevatrom MD2, Siemens, Erlangen, Germany) that supplies X-ray therapeutic beam with an energy broadband of 6 MV. The animals in the control group did not receive RT until the day they were sacrificed.

Histopathological evaluation

Following the sacrification on the postoperative day 10, en bloc excision was performed on the paws of the subjects from the metatarsal head to the middle portion of the mid-phalanx in order to preserve tissue integrity of the peritendinous adhesions. The tissue samples were embedded in paraffin blocks after decalcification with 4% hydrochloric acid and 4% formic acid solution. Longitudinal sections with a thickness of 4 microns were obtained from tissue blocks of the resected specimens. Samples were stained with hematoxylin-eosin (H&E) and examined under the light microscope. Von Gieson’s histochemical staining method was applied to the sections in order to visualize the collagen fibers in the adhesion site. The adhesions in the peritendinous area were qualitatively and quantitatively evaluated using the histological grading criteria defined by Tang et al. (Table 1). The total tendon adhesion score was determined by summing the qualitative and quantitative scores obtained from each tendon sample.

**Table 1:** Grading of adhesions using the Tang scoring scale based on quantitative and qualitative findings.

Points	Quantitative features		
0	No apparent adhesions		
1	A number of scattered filaments		
2	A large number of filaments		
3	Countless filaments		
Points	Qualitative features		
0	No apparent adhesions		
1	Regular, elongated, fine, filamentous		
2	Irregular, mixed, shortened, filamentous		
3	Dense, not filamentous		

The samples were classified into four groups as follows: A total score of 0, as no adhesion in the peritendinous tissues, 2 as a mild level of adhesion, 3 or 4 as a moderate level of adhesion, 5 or 6 as the presence of severe adhesion (Table 2).

**Table 2:** Adhesion severity based on total Tang scores.

Total score	Adhesion severity
0	None
2	Mild
3-4	Moderate
5-6	Severe

The healing response in the core tendon was graded using the parameters described by Aström and Rausing and modified by Movin et al. [5,6]. Each parameter was assessed using a 4-point scoring system, where 0 is normal, 1 is slightly abnormal, 2 is moderately abnormal, and 3 is severely abnormal. The GAG (glycosaminoglycans) content subgroup of the original scale was discarded due to poor reproducibility and increased cost (Table 3).

**Table 3:** Classification of adhesions based on total Movin scores. GAG, glycosaminoglycans

Fiber structure
Fiber arrangement
Rounding of the nuclei
Regional variations in cellularity
Increased vascularity
Decreased collagen stainability
Hyalinization
GAG content
Total semiquantitative score and general description

Statistical analysis

SPSS Version 13.0 (SPSS Inc., Chicago, IL, USA) was used for the statistical evaluation of the data. Qualitative and quantitative evaluation results were given by number and percentage. Tang and modified Movin scoring results were expressed as median, minimum, and maximum values for each group, and the scores were compared between groups using the Mann-Whitney U test. Spearman’s correlation and Mann-Whitney U tests were used to determine whether the weight and gender of the samples have an effect on the formation and level of adhesion. A probability level of p<0.05 was considered statistically significant.

## Results

Two subjects in the RT group and one subject in the control group died before the sacrification day. One paw of one rabbit from the control group was excluded from the study due to a suppurative surgical site infection. As a result of microtome sectioning and tissue damage during histochemical staining procedures, one subject from each of the control and RT groups could not be eligible for histopathological evaluation. The data obtained from the study were collected from a total of 23 slides provided from the tissues of eight rabbits in the control group, and 26 slides provided from the tissues of seven rabbits in the RT group.

**Figure 4:**
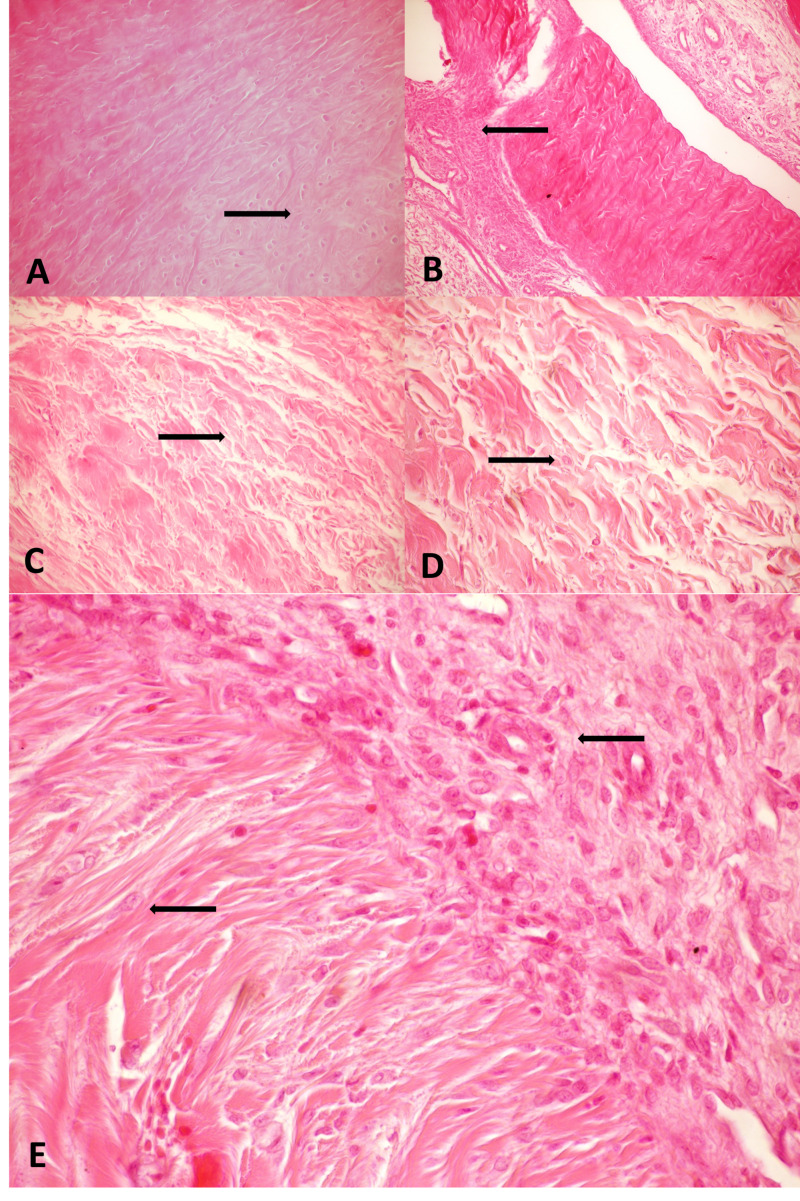
Microscopic view of the tissues obtained from rabbits in the RT and control groups. Arrows depict the defined histopathological characteristics for each microscopic view (H&E; x40). (A) Tendon from the control group: regular, elongated, fine, filamentous adhesion in the control group. (B) Tendon from the control group: longitudinal section of a tendon in the control group with mixed, short, and long filaments extending from peritendinous granulation tissue into the tendon substance (C) Tendon from the RT group: irregular, mixed, shortened filaments scattered around the amorphic hypocellular non-filamentous adhesion tissue. (D) Tendon from the RT group: fibroblast accumulation between collagen deposits. (E) Tendon from the RT group: coarse, irregular filaments extending from the vascularized peritendinous tissue into the tendon substance, with fibroblasts migrating from the peritendinous tissue into the tendon substance and adhesion tissue. RT, radiotherapy; H&E, hematoxylin-eosin

There was no significant difference between the RT and control groups in terms of gender and weight of the subjects (p>0.05). The minimum, maximum, median, and mean values of Tang scores and modified Movin scores of both groups are given in Tables 4 and 5. Peritendinous adhesion was significantly increased in the RT group compared with the control group (p<0.001) (Figure 4).

There was not a significant difference in the total quality of the healing tissue in the tendon core according to the modified Movin scoring. The rounding of the nucleus was significantly more visible in the control group (p˂0.01). We did not observe any signs of RT-related necrosis, bleeding, and apoptosis in the surrounding tissues.

**Table 4:** Tang scores of the study and the control group. *p˂0.001.

	Study group (n=26)				Control group (n=23)			
	Mean	Median	Min	Max	Mean	Median	Min	Max
Tang score	1.73	2.00*	0.00	3.00	0.70	0.00*	0.00	3.00

**Table 5:** Modified Movin scores of the study and the control group.

	Control group (n=23), mean (min-max)	Study group (n=26), mean (min-max)	p-Value
Fiber structure	2 (1-3)	2 (1-3)	0.199
Fiber arrangement	2 (1-3)	2 (1-3)	0.051
Rounding of the nuclei	2 (1-3)	1 (1-3)	0.007*
Regional variations in cellularity	1 (0-3)	1 (0-2)	0.787
Increased vascularity	0 (0-2)	0 (0-1)	0.474
Decreased collagen stainability	1 (0-2)	1 (0-2)	0.686
Hyalinization	1 (0-2)	1 (0-2)	0.557
Total semiquantitative score	10 (3-15)	8 (3-15)	0.256

## Discussion

The main goal of surgical repair of the tendons is to obtain maximum tendon strength and movement. However, injuries that occur at the level of digital synovial sheath often result in restrictive posttraumatic adhesions [7]. Despite all these efforts to prevent tendon adhesion, the results of tendon repair are still unpredictable and unsatisfactory. Treatment options such as use of physical barriers, tendon surface lubrication enhancing agents, antiadhesive pharmacological chemicals, and antimetabolites effective on cell cycle and apoptosis have been shown to be useful in the experimental studies, yet none of them is used in the management of tendon adhesions [8-10].

The effects of RT, a treatment option with ease of use, and dose standardization on the management of posttraumatic tendon adhesion have not been studied before. The main mechanism of action of RT is to prevent an inflammatory response and subsequent adverse and undesirable tissue response by provoking the formation of free radicals by the ionizing radiation and the effect of these radicals on DNA metabolism and gene transcription in the targeted tissue [11,12].

In this study, we attempted to evaluate the efficacy of an RT dose of 3 Gy on tendon healing in rabbits. We observed that RT briefly affected the inflammatory response. The histopathological evaluation of the peritendinous adhesion areas revealed increased inflammatory response and disorganized ECM structure, possibly due to inappropriate dosing. On the other hand, the roundness of the tenocyte nuclei was higher in the treatment group, suggesting a more intact and ameliorated tissue structure.

An experimental study on rats reported that endotenon response in terms of proliferation, cell surface adhesion antigens, and growth factor expression was limited in comparison to epitenon and synovial sheath on day 3 following the injury, and the release of beta-fibroblast derived growth factor (b-FGF) started to increase in the tissue structure [13]. Similarly, in an animal study on the flexor tendons of rats, it was observed that cell migration from the peritendinous tissues to the tendon tissue started on the third day after injury and reached its maximum on the fifth day [14]. Depending on the studies on the posttraumatic metabolic response, it was decided that the appropriate time for the treatment to be effective on extrinsic healing was between zero and three days following the trauma. In order to minimize the possible effects on intrinsic healing that will take place shortly after the extrinsic healing process, the administration of RT was planned on the first postoperative day. We determined the sacrification time as the end of the 10th postoperative day since animal studies on flexor tendon adhesion in the literature have shown that seven days after the partial rupture of intrasynovial flexor tendons was sufficient for the development of adhesion. In our study, we observed that the study and control groups developed posttraumatic adhesion that could be evaluated histologically by Tang scoring.

Antiproliferative effects of single-dose beta and gamma radiation in non-lethal doses on various types of human and animal tissue cells have been shown in several studies [15,16]. Liebmann et al. stated that a single dose of fractional X-irradiation applied in the acute phase of the inflammatory response suppresses the inflammatory response by deteriorating the expression of inducible nitric oxide synthase (iNOS) protein and consequently NO production by macrophages, thus restricting the inflammatory response [17]. Studies on the effect of ionizing radiation on wound healing reported decreased number of neutrophils and macrophages in the trauma site, delayed fibroblast proliferation, and migration, and deficient levels of actin polymerization following the application of radioactive ions. In their study on cell cultures produced from human Tenon capsule fibroblasts, Constable et al. reported that the number of 250, 500, 750, 1,000, and 2,000 Gy beta-irradiated fibroblasts in the culture media were significantly lower compared with the control group in every stage of the study, and a significant antiproliferative effect occurred at doses above 250 Gy [18]. They also stated that the decreased number of fibroblasts was a consequence of the deteriorated cell proliferation and cell cycle, thus the lack of apoptosis had prevented the inflammatory response after cell destruction.

It has been shown that fibroblast response can be in various patterns at different radiation doses, as low doses of beta-radiation suppress fibronectin production and high doses increase storage of collagen [19,20]. In order to find the dose range to modulate the synovial response to reduce peritendinous adhesion, it is crucial to determine the optimum dose with a higher number of subjects. Although RT is an ideal treatment method due to its positive properties such as extracorporeal use, no necessity of perioperative application, and homogeneous efficacy in the tissue, we observed that RT increased the peritendinous adhesion in the dose of 3 Gy.

Tendon healing is a multifactorial process with extrinsic and intrinsic mechanisms using intercellular mediators and cell chemotaxis. The current treatment modalities aim to prevent adhesions by affecting different elements of this process at different stages of tendon repair. Two basic theories have been proposed in the healing process of injured tendons: extrinsic healing with activated granulation tissue cells migrating from the synovial sheath and peritendinous tissues, and intrinsic healing by fibroblasts within the tendon itself originating from the endotenon and epitenon [21]. Cell culture and in vivo studies have shown that synovial tissue derived fibroblasts synthesize higher amounts of proteins required for ECM-cell communication, macrophage-fibroblast interaction, cell movement, contraction, and cell proliferation compared with endotenon and epitenon, and that synovial tissue-derived fibroblasts have a more contractile nature [22,23]. Costa et al. showed that the proliferative response to growth factors is higher in the synovial tissue than the epitenon and endotenon [24]. All these data suggest that fibroblasts with different phenotypes are activated at particular stages of the posttraumatic response in the tissue they are originated and have an individual pattern of action. The adhesion formation in the tissues treated with RT in our study group may be due to the fact that various cells in different layers of the tendon structure respond to the same radiation dose by increasing inflammatory response. Although the total semiquantitative Movin score was lower in the RT group, this finding did not reach statistical significance. However, one of the variables in the scoring system, rounding of the tenocyte nuclei score, was significantly lower, suggesting a role for RT in the prevention of tenocyte differentiation into abnormal cell morphology.

In our study, RT did not have the desired effect of preventing aberrant and unwanted soft tissue response, as opposed to its use in the treatment of heterotopic ossification and Dupuytren contracture [25-27]. A possible crucial factor differentiating the use of RT for peritendinous adhesions may be the kinetic nature of the flexor tendon. Unlike palmar aponeurosis that transforms into chords constricting the peritendinous structures in Dupuytren’s disease, or the pericapsular tissues in heterotopic ossification, flexor tendon is a very mobile tissue, constantly gliding in the synovial sheath. Immobilization of the tendon results in a dense adhesion between the tendon surface and the synovial sheath, which is biomechanically stronger than the tendon itself [28]. Among all molecules and substances applied in order to prevent adhesion, early mobilization is still the only proven method to decrease adhesion [29]. Nevertheless, research to find an agent to inhibit the adhesions should continue, as tendon lacerations may be a component of a crush injury that precludes early mobilization.

Another factor for the ineffectiveness of the RT may be the inaccurate timing of the treatment. We had hypothesized that extrinsic healing in the peritendinous tissue would begin in the first three days after trauma, and activation of the endotenon cells responsible for intrinsic healing would occur later. This assumption was based on studies evaluating the cellular response after tendon laceration performed in rats. By the time this experiment was conducted, there was no study on the synovial response in rabbits. The timing of fibroblast activation may differ between animal species, rendering the radiation on third day ineffective.

## Conclusions

To our knowledge, this is the first experimental study on the effects of RT on the tendon healing process; thus, it is not possible to draw a precise conclusion on our findings and compare the effects of RT on tendon structure with other studies on tendon injury and adhesion formation. Despite its role in the increased level of adhesion with 3 Gy irradiation in our subjects, it is essential to determine an optimal irradiation dose that would promote tendon healing and subsequently suppress the cells causing tendon adhesion with further studies.
